# Study protocol for a parallel-group, double-blinded, randomized, controlled, noninferiority trial: the effect and safety of hybrid electroconvulsive therapy (Hybrid-ECT) compared with routine electroconvulsive therapy in patients with depression

**DOI:** 10.1186/s12888-019-2320-3

**Published:** 2019-11-06

**Authors:** Han Rong, Shu-xian Xu, Jing Zeng, Ying-jia Yang, Jie Zhao, Wen-tao Lai, Li-chang Chen, Wen-feng Deng, Xiangyang Zhang, Ying-li Zhang, Min-zhi Li, Li Xiao, Xin-hui Xie

**Affiliations:** 1 Department of Psychiatry, Shenzhen Kangning Hospital, Shenzhen, Guangdong China; 2 Affiliated Shenzhen Clinical College of Psychiatry, Jining Medical University, Jining, Shandong China; 3 Center of Acute Psychiatry Service, Second People’s Hospital of Huizhou, Huizhou, Guangdong China; 4 Department of Biostatistics, School of Public Health, Southern Medical University, Guangzhou, China; 5 Laboratory of Brain Stimulation and Biological Psychiatry, Brain Function and Psychosomatic Medicine Institute, Second People’s Hospital of Huizhou, Huizhou, Guangdong China; 6 CAS Key Laboratory of Mental Health, Institute of Psychology, Chinese Academy of Sciences, Beijing, China; 7 Key Laboratory of Intelligent Information Processing, Advanced Computer Research Center, Institute of Computing Technology, Chinese Academy of Sciences, Beijing, China

**Keywords:** Study protocol, Randomized controlled trial, Hybrid electroconvulsive therapy, Electroconvulsive therapy, Depression

## Abstract

**Background:**

Electroconvulsive therapy (ECT) is the most rapid and effective treatment for patients with depression, ECT can achieve remarkable antidepressant effects in the initial 3–4 sessions, but significant side effects limit its use. However, recent low-charge electrotherapy (LCE) studies have demonstrated antidepressant or antipsychotic effects with significantly fewer side effects. The aim of this study is to propose a novel two-step charge set strategy for ECT treatment, referred to as Hybrid-ECT, to decrease side effects by using a low charge while preserving treatment efficacy.

**Methods/design:**

A randomized, double-blinded, standard-controlled, parallel-group design will be carried out. We plan to enroll 112 inpatients diagnosed with depression (unipolar or bipolar) and randomly assign them to conventional ECT (control group) or to Hybrid-ECT (treatment group, 3 ECT sessions followed by LCE sessions (approximately 2.8 joules per session)). We will evaluate participants across a wide variety of domains including clinical symptoms, cognitive, psychological and functional metrics. We will also perform magnetic resonance imaging (MRI) and event-related potential (ERPs) assessments during treatment to explore brain function differences between ECT and LCE.

**Discussion:**

This research proposes a simple but completely novel ECT strategy that aims to rapidly relieve depressive symptoms and minimize side effects. The mechanism of ECT and LCE will be further discussed.

**Trial registration:**

Chinese Clinical Trial Registry, Number: ChiCTR1900022905 (Registration date: April 30, 2019).

## Background

Globally, major depressive disorder (MDD) is one of the most common psychiatric disorders, rated as one of the five leading cause of years lived with disability (YLDs) in 2016 and associated with substantial disabilities [1]. Patients with depression often show high suicide risk [2], severe impairments in social function [3] and cognitive dysfunction [4]. According to the 1995–1999 mortality data provided by the Minister of Health of the People’s Republic of China, the mean annual suicide rate was 23 per 100 000, and there were a total of 287 000 suicide deaths per year [5]. Therefore, it is especially important to quickly and effectively treat depression patients.

Electroconvulsive therapy (ECT) administered with anesthesia and neuromuscular blockers is the most effective treatment for depression [6–9]. However, the use of ECT is impeded by adverse effects (AEs), such as acute headache, dizziness and confusion [10], especially the cognitive function impairments, which may be affected by the stimulus intensity, number of treatments or electrode placement [11, 12]. Thus, researchers have been working on methods to quickly relieve symptoms while minimizing side effects.

One important feature of ECT is the rapid antidepressant effect in the early stage of treatment. Kellner et al. conducted a double-blind, controlled trial to compare the efficacy and cognitive effects of three electrode placement methods in ECT [13]. The results demonstrated that all placements resulted in rapid remission over the early course of treatment; during the first 3–4 ECT sessions, the bitemporal placement resulted in a more rapid decrease in symptom ratings than the other placements, followed by small changes in symptom ratings in the subsequent sessions for all placement groups. Some articles have reported that patients could go into remission after the first ECT session [14–16]. The findings indicated that the decline in symptom rating of later stage was not as rapid as the early period; furthermore, as the number of ECT sessions increased, the side effects became more pronounced [17], which suggests that we should change the mode of ECT procedure.

At present, based on existing ECT knowledge, a charge-induced seizure is considered to be a key feature. However, after reviewing the literature, we found an interesting phenomenon. Some ECTs that failed to induce seizures also demonstrated antidepressant effects but without severe side effects [18–20]. The safety and efficacy of low-charge nonconvulsive electrotherapy (NET) for MDD was first studied in detail by Regenold et al. in 2015 [21]. The authors demonstrated that the therapeutic effect of NET in patients with resistant MDD was similar to that of ECT, while serious AEs were not observed. Although the study was small, unblinded, nonrandomized and uncontrolled by a sham or standard procedure, and the results were susceptible to both investigator and subject bias, they exhibited the potential of low-charge electrotherapy (LCE) for mental diseases and raised the following question: are high charges or seizures essential? Subsequently, a double-blind, randomized, controlled pilot clinical trial with schizophrenia patients was conducted by our team; the results suggested that LCE (2.8 joules per session) without seizures exerted similar antipsychotic effects while causing fewer AEs than ECT [22]. Although the sample sizes have been small, LCE has the potential to be a safe and effective treatment for patients with schizophrenia or depression. However, our inhouse data (detailed information please see Additional file 3: Table S2 and Additional file 4: Figure S1) revealed that LCE relieved depression more slowly in the first few sessions than ECT.

Considering that patients with depression need quick symptom relief with few side effects, we propose a new two-step charge set strategy for ECT treatment, referred to as Hybrid-ECT. In Hybrid-ECT, conventional ECT is conducted during the first 3 sessions (the first step) because according to Kellner’s research, the Hamilton Rating Scale for Depression (HAMD-24) score reduction trajectory is steep during the first 3 ECT sessions (an approximation based on Figure 2 of Kellner’s article [13]), and then LCE is conducted until the treatment is terminated (the second step). We hope that the two-step Hybrid-ECT could combine ECT’s strength of an early rapid antidepressant effect and LCE’s advantage of fewer side effects. Therefore, our hypotheses were as follows: 1) Hybrid-ECT exerts a noninferior antidepressant effect relative to ECT over the whole course of the treatment; and 2) compared with the routine ECT charge set strategy, Hybrid-ECT may cause fewer AEs, especially in the later part of treatment (i.e., after 3 ECT sessions). To test these hypotheses, we designed the present randomized, double-blinded, parallel-group, controlled clinical trial. Since the intervention arm of this trial is a change in the current ECT charge setting strategy, routine ECT was selected as the standard-control group; and the trial is designed as a noninferiority study on the antidepressant effects of Hybrid-ECT and ECT.

## Methods/design

### Study design

**Fig. 1:**
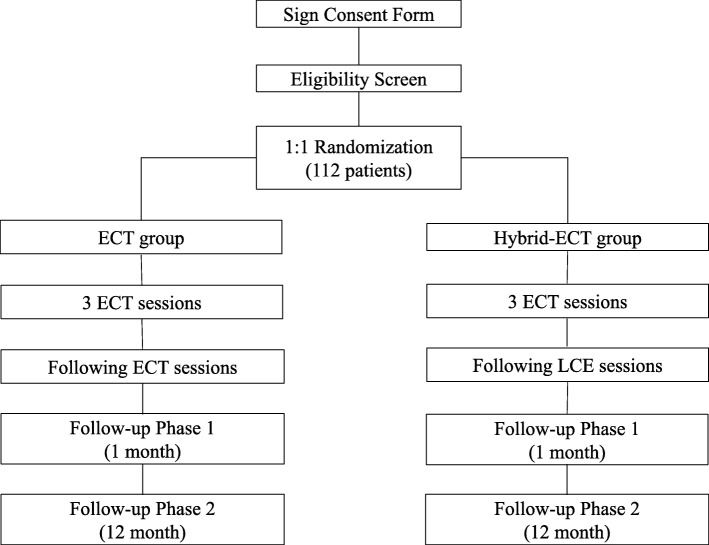
Study flow chart

This clinical trial is a randomized, double-blinded, parallel-group, standard-controlled, noninferiority study to detect the efficacy and side effects of a novel ECT charge setting strategy called Hybrid-ECT for the treatment of patients with depression compared to routine ECT. Participants, neuropsychological measurement raters, magnetic resonance imaging (MRI) and event-related potential (ERP) operators, patient psychiatrists, nurses, and researchers will be blinded to the patient treatment assignment. After each ECT/LCE procedure, the ECT operator will reset the energy parameters to prevent others from detecting the participant’s group. The study flow chart is shown in Fig. 1.

### Study population and recruitment

A total of 112 inpatients with unipolar or bipolar depression will be recruited by psychiatrists in Shenzhen Kangning Hospital (Mental Health Center of Shenzhen city, which has more than 1000 beds) in Shenzhen city, Guangdong Province, PR China.

### Inclusion criteria

1. Patient (18 < age < 60) accepted ECT for treatment because of severe depression and on a stable psychotropic medication regime more than 2 weeks;
2. Patient met the following ICD-10 criteria [23] for diagnosis of unipolar or bipolar depression (F31.4, F31.5, F32, F33);
3. Patient had an HAMD-24 score > 21;
4. Patient was competent to provide informed consent; and.
5. Patient was sufficiently fluent in Chinese to ensure valid responses to the neuropsychological tests.

### Exclusion criteria

1. Failure to respond to earlier ECT;
2. ECT within the last 6 months;
3. Rapid-cycling or mixed-episode bipolar disorder;
4. Medication use incompatible with ECT treatment (such as lithium, benzodiazepine, antiepileptic drugs, etc.). Use of these drugs will be stopped at least 5 half-lives before the start of treatment;
5. Inability to follow the study protocol;
6. Unstable, serious comorbid medical condition (Parkinson’s disease, multiple sclerosis, stroke, alcohol use disorder, etc.) or history of epilepsy;
7. Intelligence quotient (IQ) ≤ 70 tested by the Wechsler Adult Intelligence Scale 4th edition [24];
8. ≤ 9 years of education;
9. Pregnancy or women without adequate contraception; and.
10. Young Mania Rating Scale (YMRS) score [25] > 20.

### Withdrawal criteria

The patient will be withdrawn and unblinded at any time if the exclusion criteria are met, the psychiatrist finds that the patient could be better served with other treatments, there is a decrease in the HAMD-24 score of < 3 points between the latest two visits, or the patient decides to withdraw. When a patient withdraws his or her consent, the reason and withdrawal date will be recorded.

### Interventions

#### ECT group (control group)

##### Anesthesia

Patients will be administered etomidate for anesthesia, succinylcholine as a muscle relaxant, and atropine for the suppression of gland secretions, and pure oxygen will be supplied during each treatment session.

##### Placement of stimulation electrodes

Because bitemporal electrode placement achieved a more rapid and earlier decrease in symptom ratings [13], bitemporal electrode placement will be used on each patient. For the bitemporal electrode placement, the two electrodes are applied 2–3 cm above the midpoint of the line connecting the outer canthus of the eye and the external auditory meatus on each side of the head [13].

##### Stimulus and seizure threshold (ST) titration

The ECT or LCE procedure will be administered with a spECTrum 5000Q ECT instrument (MECTA Corporation, OR, USA) three times a week. The pulse width is set to 1 ms, and the current is set to 800 mA. The ST titration procedure basically follows Mankad et al. [12]. The energy set of the first ECT session uses the titration procedure described in the Additional file 1: Table S1. If the seizure induction is unsuccessful or the seizure lasts less than 15 s (based on electroencephalogram (EEG)), the patient will be restimulated at a higher energy level after a short delay. The restimulation process could be repeated up to three times during the first treatment. If all four stimulations fail to induce an adequate seizure, the dose set for the titration in the next session will be set at 2 steps higher than the last stimulation dose. The ECT charge dose for subsequent treatments is set as follows: 1.5 * ST, similar to Kellner et al. [13].

##### Seizure adequacy

Seizure detection is performed using a single channel EEG from the ECT instrument. The seizure duration recorded by EEG is checked by a certified electroencephalographer who is blinded to the participant’s status.

#### Hybrid-ECT group (treatment group)

##### Anesthesia

Same as the ECT group.

##### Placement of stimulation electrodes

Same as the ECT group.

##### Stimulus

The energy set of the first three sessions is the same as that in the ECT group; the subsequent sessions use LCE, and the energy is set to the minimum (2.8 J) with a stimulus duration = 0.5 s and a frequency = 20 Hz, as we have previously reported [22].

##### Seizure adequacy

Same as the ECT group.

##### Follow-up scheme

To evaluate the acute and chronic influence of ECT and Hybrid-ECT respectively, we set a two-stage follow-up schedule (see Table 1). The first follow-up phase is set at the one-month after the last ECT/LCE session, and the second phase is a one-year follow-up following the first phase (see Table 1).

**Table 1:**
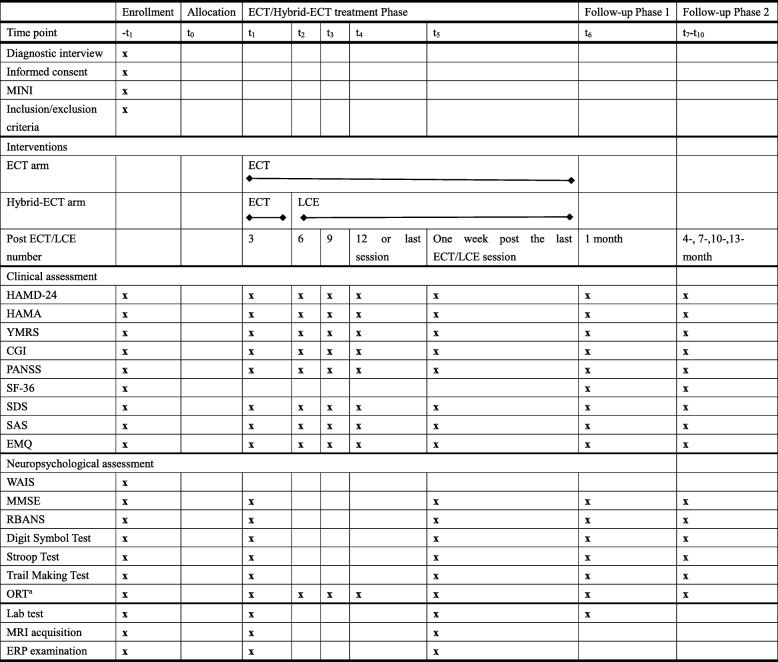
Visit procedure

a: test after every ECT/LCE session

HAMD-24: Hamilton depression rating scale, *HAMA* Hamilton anxiety rating scale, *CGI* Clinical global impressions, *YMRS* Young mania rating scale, *PANSS* Positive and negative syndrome scale, *SF-36* MOS 36-item short-form health survey, *SDS* Self-rating depression scale, *SAS* Self-rating anxiety scale, *EMQ* Everyday memory questionnaire, *WAIS* Wechsler adult intelligence scale 4th edition, *MMSE* Mini-mental state examination, *RBANS* Repeatable battery for the assessment of neuropsychological status, *ORT* Orientation recovery test

##### Randomization

After receiving detailed information about this trial, signing the consent form, passing the eligibility screen and completing the baseline test, the enrolled patients will be assigned a sequential patient number. If a patient discontinues from the study between the randomization and the first ECT procedure, the patient will be excluded from the final analyses, and he or she will not be allowed to rejoin the study.

A statistician with no other connection to the trial will generate the randomization sequence using the default random number generator of SAS version 9.4 (SAS Institute Inc., Cary, NC, USA). The randomization sequence list will remain concealed from the investigators. A research nurse who will not participate in the clinical evaluation will assign the participants to their group.

Participants, neuropsychological measurement raters, MRI and ERP operators, patient psychiatrists, nurses, and researchers will be blinded to the patient treatment assignment. If unblinding is determined to be necessary, the investigators must report all unblinding (with reason) on the corresponding case report form (CRF).

##### Assessment of the effectiveness of the blind methods

Participants and raters will be asked to which group the participant had been allocated, and about the strength of confidence of their guess as well as the reasons for their guess at every visit after the randomization. The ratios of right guesses/wrong guesses will be used to assess the effectiveness of the blinding procedure.

##### Medication during ECT/hybrid-ECT sessions

Patients in both arms will be maintained on their previously prescribed antidepressants and antipsychotics (usually used for patients with bipolar depression and patients with depression and psychotic symptoms) during the trial. Anticonvulsant drugs, lithium, or mood stabilizers will be discontinued during the course of ECT/Hybrid-ECT treatment. Patients exhibiting agitation or feeling anxious could be temporarily administered short half-life benzodiazepines, but the administration of benzodiazepines will be prohibited 24 h before the ECT/LCE session. When patients suffer from insomnia, zopiclone, eszopiclone, or zolpidem can be temporarily given to help them sleep.

##### Medication during the follow-up phase

Medication during the follow-up period is essentially the same as that during the ECT/Hybrid-ECT phase; however, lithium, mood stabilizers, or antiepileptic drugs are allowed. The detailed and individualized pharmacological plan will be determined by the patient’s psychiatrist.

### Outcome measures

#### Visit procedure

Patients will be interviewed during twelve visits. The detailed visit procedure is shown in Table 1.

#### Primary outcome measure

The primary outcome measure is the change in total HAMD-24 score at endpoint (visit t_5_) from baseline.

#### Secondary outcome measures

The secondary outcome measures include the response rate (defined as a HAMD-24 score that shows a more than 50% reduction from the baseline) and remission rate (remission is defined as HAMD-24 score < 10) at t_5_ and the scores on the Clinical Global Impressions (CGI) scale [26], Positive and Negative Syndrome Scale (PANSS) [27], MOS 36-item Short-form Health Survey (SF-36) [28], Hamilton Anxiety Rating Scale (HAMA) [29], YMRS [25], Self-Rating Depression Scale (SDS) [30], Self-Rating Anxiety Scale (SAS) [31], and Everyday Memory Questionnaire (EMQ) [32].

#### Neuropsychological assessment

A comprehensive neurocognitive battery will be performed, as presented in Table 1. Neuropsychological evaluations will be carried out by trained psychologists who will be blinded to the patient groups. The cognitive domains to be tested include general intelligence, global status, attention, processing speed, language, visuospatial function, executive function and memory. IQ will be assessed by the Wechsler Adult Intelligence Scale 4th edition [24]. A modified version of the Mini-Mental State Examination (MMSE) [33] will be used to test global status. The Repeatable Battery for the Assessment of Neuropsychological Status (RBANS) is designed to assess attention, language, visuospatial, immediate memory and delayed memory [34]. Processing speed will be assessed by the digit symbol test [35]. Stroop reaction time, Stroop color-word test [36] and trail making test (A, B) [37] will be used for examination of executive function. Orientation recovery is assessed at every ECT/LCE session. The time to orientation test is assessed with continuous questioning on five items (name, place, day, age, date of birth) for 90 min [38]. A correct response on four of five items is required. Patients who do not meet the criteria for recovery are assigned scores of 100 min. The subjective cognitive evaluation will be evaluated by the Squire Subjective Memory Questionnaire [39].

#### MRI and ERP acquisition

ECT can cause changes in brain function [40], metabolism [41], and structure [42], especially in hippocampal volume [43], which may be related to neurogenesis in the hippocampal dentate gyrus, so the aims of our MRI and ERP are as following: 1) use the MRI to observe the changes of hippocampus in volume before and after treatment and between the two groups; 2) use the fMRI and DTI to observe ECT/LCE-related activation, functional connections, and changes in nerve fiber connections; 3) use MRS to observe metabolic changes of hippocampus before and after ECT; 4) use ERP to observe the EEG changes related to ECT/LCE.

#### MRI protocol

MRI will be performed at 3 time points: t_0_ (~ 24–48 h before baseline), t_1_ (~ 24–48 h after the three first ECT and before the fourth ECT/LCE) and t_5_ (~ 7 days the last of ECT/LCE). All participants will be scanned at the Shenzhen Kangning Hospital Neuropsychiatry Imaging Center in a 3 T Discovery MR750 scanner (GE Healthcare, Milwaukee, USA) equipped with an eight-channel head coil. A standard MRI protocol will be applied at each time point, including a T1-weighted spoiled gradient echo, SPGR (TE = min full; TR = 6.7 ms; flip angle = 12°; field of view =256 mm; voxel size = 1.0 * 1.0 * 1.0 mm^3^; matrix = 256 * 256); a T2-weighted high resolution hippocampus sequence (TE = 50 ms; TR = 8020.0 ms; flip angle = 122°; field of view =175 mm; voxel size = 0.4 * 0.4 * 2.0 mm^3^; matrix = 448 * 448); a resting-state fMRI scan with a BOLD sequence (TE = 25 ms; TR = 2000 ms; field of view =220 mm; voxel size = 3.4 * 3.4 * 3.2 mm^3^; matrix = 64 * 64; volume number = 240); a DTI sequence (TE = 81.4 ms; TR = 8724.0 ms; field of view = 224 mm; voxel size = 2.0 * 2.0 * 2.0 mm^3^; matrix = 112 * 112; number of diffusion directions = 64; b values = 0, 1000 s/mm^2^); ^1^H- magnetic resonance spectroscopy (MRS) sequences for the ROIs including the hippocampus, thalamus, anterior cingulate and posterior cingulate (TE = 35.0 ms; TR = 1500 ms) [44–46]; and a 3D ASL sequence (TE = 10.5 ms; TR = 4389 ms; post-labeling delay = 1025 ms; field of view =240 mm).

#### ERP measures

The ERP data will be recorded using BrainAmp Amplifier (Brain Products GmbH, Zeppelinstraβe 782,205 Gilching, Germany), and electrodes will be placed at 32 scalp locations based on the 10–20 system [47]. An electrode linked ears/mastoid reference scheme will be used. The electrode impedance will be kept below 10 KΩ. Amplifiers will have a bandpass of 0.1–50 Hz, and the stimulus rate will be 0.5 Hz. Continuous EEG records, at a sampling rate of 1000 Hz, will be stored for further off-line analysis. In the ERP experiment, the patients will be told to stay relaxed and quiet. P300 and P50 paradigms and 3-min resting-state EEG recording with eyes closed will be selected for the measurement index of cognitive function. The classic auditory oddball paradigm will be used to measure P300. The classic P300 task will include a total of 200 target and standard stimuli, and the 40 target stimuli will be a tone at 60 dB of low frequency (20%) and high pitch (2000 Hz), and the 160 standard stimuli will be a tone at 80 dB of high frequency (80%) and low pitch (1000 Hz). The two stimuli presentations last 20 ms. The participant will need to click a button as soon as they hear the stimuli to judge whether a tone was a standard stimulus or target stimulus [48, 49]. P50 will be examined using conditioning (S1)-testing (S2) paired acoustic stimulation with short wavelength and sound pressure set at 105 dB. The first click produces an excitatory response and activates inhibitory pathways, and the response to the second click is normally suppressed. The pairs of clicks will be presented 500 ms apart and the interval between two paired stimuli is 10 s [50, 51]. P50 is measured by the P50 ratio, which will be calculated as the S2 amplitude divided by the S1 amplitude. A P50 ratio closer to 0 is indicative of robust suppression (gating), whereas a ratio closer to 1 is indicative of diminished sensory gating [52]. The ERP procedures will be performed according to the MRI schedule.

#### Safety assessment and AEs

Safety data will include clinical examinations, blood tests, electrocardiogram (ECG), urine tests, and cognitive function assessments.

#### AEs

Any untoward medical occurrence during ECT/Hybrid-ECT treatment, which may or may not be causally linked with the ECT/LCE treatment, will be defined as an AE. All AEs will be recorded in the CRF. An important focus of this trial is to explore whether there are fewer side effects from Hybrid-ECT than from routine ECT, in addition to comparing total AE numbers and severity; therefore, all AEs will be categorized according to when they occurred (initial three ECT sessions or the subsequent ECT/LCE sessions) for further analyses.

#### Sample size and power calculation

Only one study [21] has measured changes in depression symptom and cognitive effects between pre- and post-NET. Therefore, to test the first hypothesis, we used noninferiority tests [53] calculated using the Power Analysis and Sample Size Software (ver.15.0) (NCSS, LLC. Kaysville, Utah, USA) for the difference between two means (noninferiority margin = 4.0, standard deviation (SD) = 8.0, power = 0.8, a = 0.05 (one-tailed), drop-out rate = 10%) of the change in HAMD-24 scores with a 1:1 allocation, and we obtained a total sample size of 112 (56 for each arm).

### Statistical analyses

#### Neuropsychological and clinical outcomes

Demographic and baseline data will be analyzed using independent two-sample *t*-tests or chi-square tests (or Fisher’s exact tests). All analyses of clinical outcomes, neuropsychological results and safety indicators will be performed for the modified intent-to-treat population (MITTP) (at least one treatment and at least one postbaseline measure). We will use mixed-effects model repeated measures (MMRM) as the main analysis for the comparison of the primary and continuous secondary outcome measures. We will use chi-square/Fisher’s exact tests to analyze the categorical outcome measures. We will use a “tipping point” analysis to assess the robustness of the primary analysis (MMRM) to possible deviations from the missing at random (MAR) assumption. The significance level will be set to 0.05 (two tailed). All statistical analyses will be performed with SAS (ver. 9.4) software (SAS Institute Inc., Cary, NC, USA). MRI and ERP data analyses will be performed by using the relevant professional software packages (FreeSurfer [54], FSL [55], EEGLAB [56], etc.*)*.

## Discussion

### Summary

To the best of our knowledge, this is a completely novel energy set strategy for ECT procedures. We speculate that Hybrid-ECT may rapidly relieve depressive symptoms during the early stage, then we will convert to LCE until the symptoms are completely relieved, and the side effects will occur less often than those in the control group (routine ECT). Furthermore, we will also perform the MRI and ERP assessments before and after the ECT treatment to test the hypothesis that Hybrid-ECT and routine ECT share the same antidepressant mechanisms. The strengths of the study include an innovative experimental design, time-intensive visits, detailed neuropsychological assessments, and MRI and ERP examinations to accurately document the differences in efficacy and side effects between Hybrid-ECT and ECT.

### Limitations

Potential weaknesses include a relatively small sample size and the expected heterogeneity in the patients’ use of medications, both of which may increase the variance in the study measures. Furthermore, we lack a third arm using a sham ECT to further assess the potential therapeutic efficacy of Hybrid-ECT, but this task has proven difficult to accomplish due to the ethical concerns of exposing patients to anesthesia when there is no expectation of a clinical benefit. Therefore, we chose routine ECT as the standard control. A larger multi-center trial is required, along with methods for measuring biomarkers to better understand and regulate the application of Hybrid-ECT in the treatment of depression.

### Study feasibility

Shenzhen Kangning Hospital is the largest psychiatric hospital in Shenzhen city, treating thousands of patients with depression every year. Our hospital has been conducting ECT for many years without serious medical accidents. The researchers have rich clinical and scientific experience, and we have published a relevant pilot clinical trial [22], which led us to design Hybrid-ECT to further reveal the secrets of ECT.

### Perspectives

If Hybrid-ECT is found to rapidly relieve depressive symptoms with fewer side effects, this could have important implications for future ECT strategies in treating patients with depression and the scientific understanding of the mechanisms of ECT-related cognitive decline in patients. In addition, this study may improve the understanding of ECT-related antidepressant mechanisms.

### Trial status and dissemination

Participant enrollment will be initiated approximately in July 2019 and is expected to be completed by July 2020. The findings of this study will be published in peer-reviewed scientific journals and presented at scientific conferences. The SPIRIT-Checklist please see the Additional file 2.

## Supplementary information

**Additional file 1: Table S1.** Charge titration procedure.

**Additional file 2.** SPIRIT-Checklist.

**Additional file 3: Table S2.** Results of preliminary trial.

**Additional file 4: Figure S1.** The HAMD Change of preliminary trial.

## Data Availability

A staff member will be appointed as a data administrator. Patients’ data include electronic files and paper documents. The paper document will be stored in locked cabinets. The electronic files will be back up on the computers and be password and firewall protected. A data monitoring committee (DMC) within the good clinical practice (GCP) office in the Kangning Hospital has been established. The DMC is independent of the present trial staff and has the right to access and audit any data during the trial. The investigators and statistician will allow access to the final trial dataset after the trial is finished, and the participants will have access to his or her own data. As the sample size is moderate (112 patients), interim analyses will not be performed. The blood and stool samples and the MRI and ERP data will be stored for future use in ancillary studies. We will use a data-driven algorithm or machine learning algorithm to further detect the relationships between clinical outcomes and biological measures. The clinical data without personal information will be shared on the registration center after the trial finished.

## Abbreviations

AE: Adverse effects
CGI: Clinical Global Impressions
CRF: Case report form
DMC: Data monitoring committee
ECT: Electroconvulsive therapy
EEG: Electroencephalogram
EMQ: Everyday Memory Questionnaire
ERPs: Event-related potentials
GCP: Good clinical practice
HAMA: Hamilton Anxiety Rating Scale
HAMD-24: Hamilton Rating Scale for Depression
Hybrid-ECT: Hybrid electroconvulsive therapy
IQ: Intelligence quotient
J: Joule
LCE: Low-charge electrotherapy
MAR: Missing at random
MDD: Major depressive disorder
MITTP: Modified intent-to-treat population
MMRM: Mixed-effects model repeated measures
MMSE: Mini-Mental State Examination
MRI: Magnetic resonance imaging
MRS: Magnetic resonance spectroscopy
NET: Nonconvulsive electrotherapy
PANSS: Positive and Negative Syndrome Scale
RBANS: Repeatable Battery for the Assessment of Neuropsychological Status
SAS: Self-Rating Anxiety Scale
SD: Standard deviation
SDS: Self-Rating Depression Scale
SF-36: MOS 36-item Short-form Health Survey
ST: Seizure threshold
YMRS: Young Mania Rating Scale
