# Supplementary material for: Study protocol for a parallel-group, double-blinded, randomized, controlled, noninferiority trial: the effect and safety of hybrid electroconvulsive therapy (Hybrid-ECT) compared with routine electroconvulsive therapy in patients with depression
Source: BMC Psychiatry. 2019 Nov 6;19:344. doi: 10.1186/s12888-019-2320-3 (PMC6836661; doi:10.1186/s12888-019-2320-3)
Supplement: Supplementary file 1 — Additional file 1: Table S1. Charge titration procedure. [file 12888_2019_2320_MOESM1_ESM.docx]

STable 1. Charge titration procedure

| Dose level | Frequency (Hz) | Duration (s) | Energy (Joule) | Charge (mC) |
| --- | --- | --- | --- | --- |
| 1 | 40 | 0.5 | 5.6 | 32 |
| 2^a^ | 40 | 0.75 | 8.4 | 48 |
| 3^b^ | 40 | 1.25 | 14.1 | 80 |
| 4 | 40 | 2.00 | 22.5 | 128 |
| 5 | 60 | 2.00 | 33.8 | 192 |
| 6 | 60 | 3.00 | 50.7 | 288 |
| 7 | 60 | 4.50 | 76.0 | 432 |
| 8 | 60 | 6.00 | 101.4 | 576 |

a: Start at level 2 in female patients;

b: Start at level 3 in male patients.
