# Supplementary figures and images for: Study protocol for a parallel-group, double-blinded, randomized, controlled, noninferiority trial: the effect and safety of hybrid electroconvulsive therapy (Hybrid-ECT) compared with routine electroconvulsive therapy in patients with depression
Source: BMC Psychiatry. 2019 Nov 6;19:344. doi: 10.1186/s12888-019-2320-3 (PMC6836661; doi:10.1186/s12888-019-2320-3)

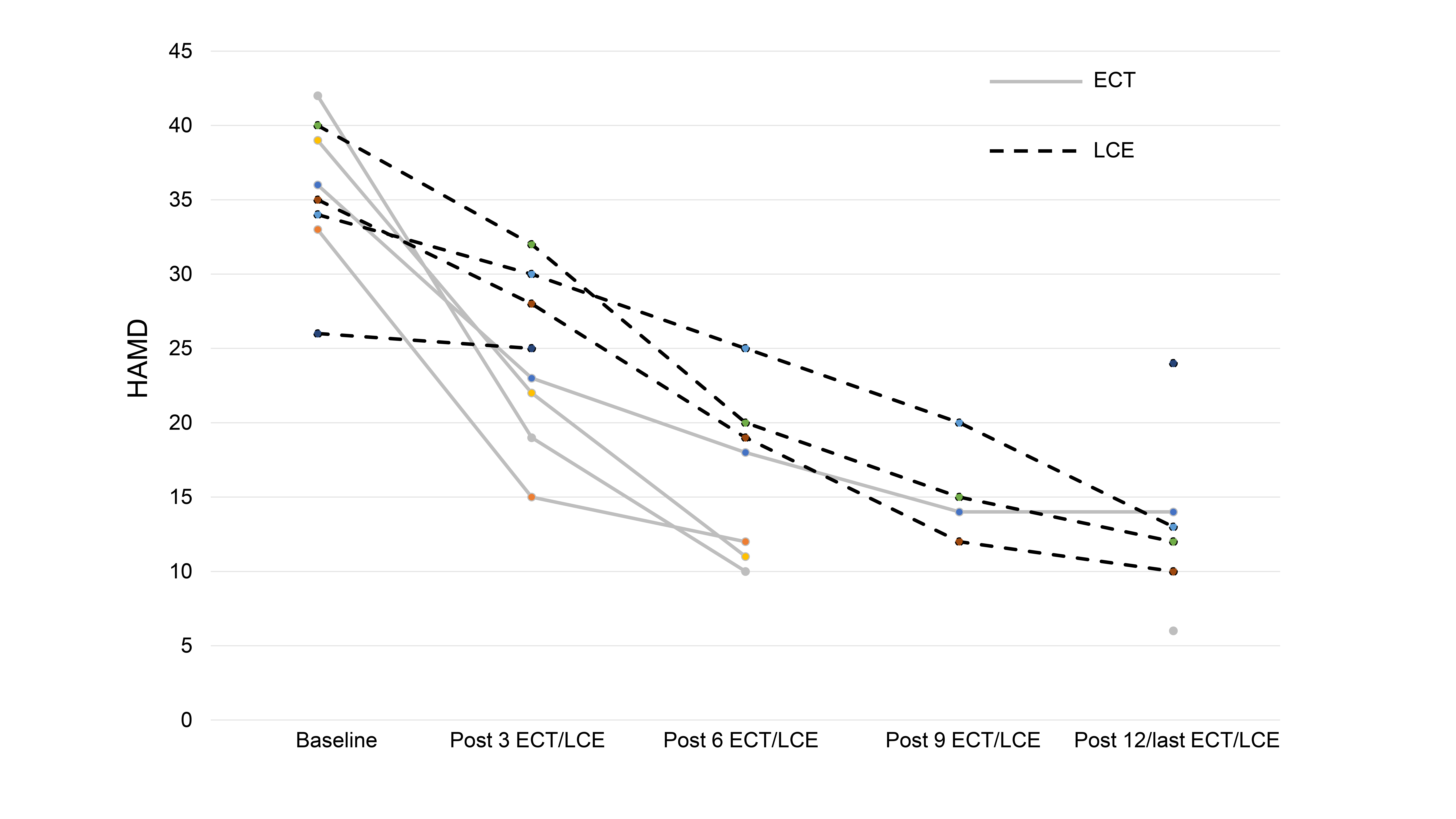

Supplement: Supplementary file 4 — Additional file 4: Figure S1. The HAMD Change of preliminary trial. [file 12888_2019_2320_MOESM4_ESM.tif]
